# Dr. S. P. Hullihen's Compound Screw Forceps

**Published:** 1849-04

**Authors:** 


*Dr. S. P. Hullihen's Compound Screw Forceps.*—In the Eureka, a record of mechanism, inventions, patents, science and news, for Wednesday, April 18th, we find an engraving, and description of Dr. S. P. Hullihen's compound screw forceps, which, as would seem from the above named publication, was patented by C. H. Dubs, of Natchez, Miss. October 17th, 1848. Now this instrument was invented several years ago by Dr. Hullihen of Wheeling, Va., and a description, accompanied by an engraving, was published soon after, in the American Journal of Dental Science, and in the writer's Principles and Practice of Dental Surgery, so that it would seem almost impossible, that Mr. D. could have been ignorant that Dr. Hullihen was the inventor, and if so, he has been guilty of the most barefaced fraud. He may, perhaps, claim that his instrument is different, but the general construction and principle upon which the two instruments act, is precisely the same, although the conical screw between the jaws of the instrument is worked somewhat differently, but the very slight alteration here can scarcely be called an improvement. In exposing this fraud, we have felt ourselves called upon to do so by a sense of justice to the profession, as well as to Dr. Hullihen, the gentleman to whom the credit of this really valuable invention truly belongs.

